# Outcomes of transarterial chemoembolization plus percutaneous radiofrequency ablation for early-stage hepatocellular carcinoma with tumor diameter > 3 cm versus ≤ 3 cm: a single-center retrospective study

**DOI:** 10.3389/fonc.2026.1727692

**Published:** 2026-03-09

**Authors:** Yong Xie, Tianshi Lyu, Haitao Guan, Li Song, Yinghua Zou, Jian Wang

**Affiliations:** Department of Interventional Radiology and Vascular Surgery, Peking University First Hospital, Beijing, China

**Keywords:** early-stage, hepatocellular carcinoma, progression-free survival, radiofrequency ablation, transarterial chemoembolization

## Abstract

**Background/objective:**

To investigate the effectiveness and safety of transarterial chemoembolization (TACE) plus percutaneous radiofrequency ablation (pRFA) (TACE-pRFA) for very early/early-stage hepatocellular carcinoma (HCC) with tumor diameters >3 cm versus ≤ 3 cm.

**Methods:**

In this retrospective study, we enrolled 118 patients who underwent TACE-pRFA for a single HCC (≤ 5 cm) from February 2014 to December 2021. Patients were divided into two groups according to the maximum tumor diameter (≤ 3 cm versus > 3 cm). Regular follow-up was conducted after pRFA to assess progression-free survival (PFS). TACE-pRFA-related complications were evaluated. Univariable and multivariable Cox proportional-hazards regression analyses were performed to identify risk factors for PFS.

**Results:**

The median PFS of the total cohort was 35.0 months (95% confidence interval [CI], 24.3–45.7). The 1-, 3-, and 5-year cumulative PFS rates in the whole cohort were 84.7% (95% CI, 78.1–91.9%), 47.5% (95% CI, 38.2–59.0%), and 31.5% (95% CI, 22.2–44.9%), respectively. Univariable and multivariable analyses showed that the maximum tumor diameter (p = 0.023) was an independent prognostic factor for PFS after TACE-pRFA. Treatment-related complications were comparable between the ≤ 3 cm group and the > 3 cm group.

**Conclusion:**

TACE combined with pRFA is safe for very early/early-stage single HCC ≤5 cm. Maximum tumor diameter is an independent prognostic factor for PFS, while treatment-related complications are comparable between tumors ≤3 cm and >3 cm.

## Introduction

A variety of locoregional therapies are widely used for treating hepatocellular carcinoma (HCC). Since percutaneous radiofrequency ablation (pRFA) is less invasive, more cost-effective, and associated with fewer postoperative complications, it is recommended by the Barcelona Clinic Liver Cancer (BCLC) guidelines as an effective treatment for patients with very early or early-stage HCC (1). pRFA can achieve complete cure of HCC with a diameter of ≤ 3 cm, which is widely recognized internationally (2, 3). However, the main challenge of pRFA is frequent tumor progression after ablation, which is directly associated with patient prognosis. The impact of tumor location or size on pRFA effectiveness remains controversial. Some recent studies have reported that the location (e.g., subcapsular versus non-subcapsular) and diameter of HCC can affect the safety and efficacy of pRFA (3, 4). Obtaining a sufficient safe ablation margin may address these issues. Thus, combination therapy with transarterial chemoembolization (TACE) and pRFA (hereafter referred to as TACE-pRFA) was initially proposed as a strategy to achieve a larger ablation zone, as it can reduce perfusion-mediated heat loss (5). Subsequently, numerous studies have been conducted to verify its practical utility (6).

Currently, the combination of TACE and pRFA has been reported to yield favorable outcomes in the treatment of small HCC (3, 4, 7–9). However, research on risk factors for tumor progression remains limited, and findings have varied across studies. For example, several retrospective studies have confirmed multiple risk factors for tumor progression or recurrence, such as tumor size, specific tumor location, and alpha-fetoprotein (AFP) level (4, 10). In contrast, previous studies have identified serum albumin level, hepatitis B infection, and treatment modality as important independent prognostic factors for tumor progression (7, 11). Therefore, further research is warranted, as the independent prognostic factors for tumor progression following TACE-pRFA treatment for very early/early-stage HCC remain unclear.

In this study, we aimed to investigate the effectiveness and safety of TACE-pRFA in patients with very early/early-stage HCC (tumor diameter: > 3 cm versus ≤ 3 cm) and to identify the risk factors for progression-free survival (PFS).

## Materials and methods

### Participants and study design

A retrospective study was conducted on patients with very early- or early-stage HCC who underwent TACE-pRFA as first-line treatment at our institution between February 2014 and December 2021. This study was approved by the institutional review board (IRB) of our hospital. Given the retrospective design of the analysis, the requirement for written informed consent from patients was exempted.

**Figure 1:**
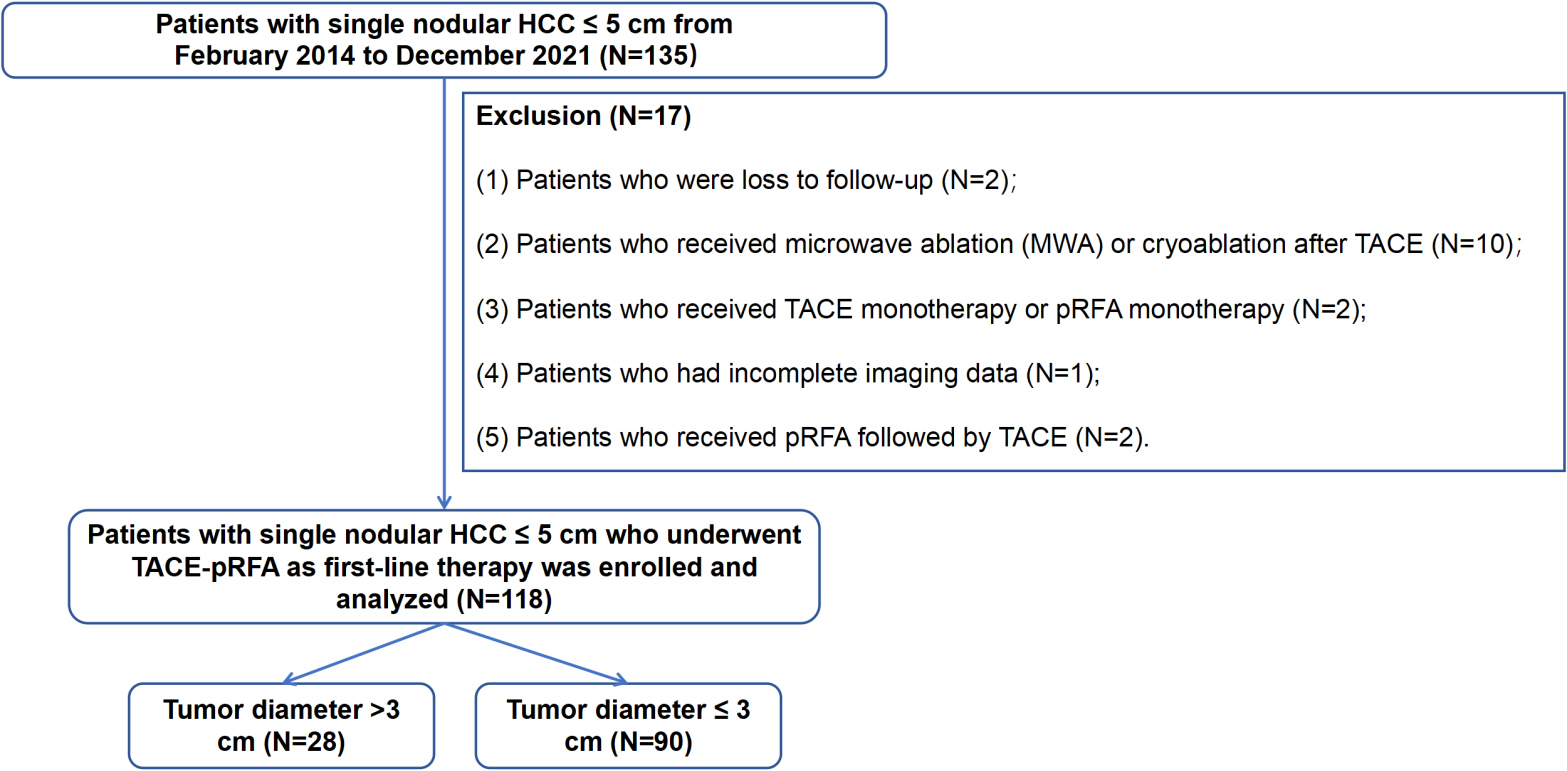
Flow chart of this study. TACE, transarterial chemoembolization; pRFA, percutaneous radiofrequency ablation; HCC, hepatocellular carcinoma.

The inclusion criteria for this study were as follows: (1) diagnosis of HCC confirmed by histopathology or in accordance with the guidelines of the European Association for the Study of Liver (EASL) and the American Association for the Study of Liver Disease (AASLD) (12); (2) single tumor lesion: ≤ 2 cm (very early-stage HCC) or > 2 cm and ≤ 5 cm (early-stage HCC); (3) age ≥ 18 years; (4) Child-Pugh class A or B; (5) Eastern Cooperative Oncology Group performance status (ECOG PS) 0–1. The exclusion criteria were shown in **Figure 1**. A total of 118 patients were finally enrolled and analyzed, including 90 patients in the ≤ 3 cm group and 28 patients in the > 3 cm group (**Figure 1**).

### TACE procedure

Under digital subtraction angiography (DSA) guidance, a 5-Fr Rosch hepatic catheter (Cook Inc., Bloomington, IN, USA) was used to cannulate the hepatic artery or perform superior mesenteric arteriography for tumor blood supply evaluation, followed by superselection of tumor-feeding arteries via a 1.9-Fr microcatheter (Asahi Intecc Co., Ltd., Japan). Dynamic contrast-enhanced cone beam computed tomography (CBCT) and three-dimensional (3D) reconstruction were conducted through the microcatheter to identify the blood supply of the lesion, with the operator paying attention to abnormally tumor-stained nodules; microcatheters were used for embolization when possible.

An emulsion was made by mixing 5–10 mL of lipiodol (Guerbet, Villepinte, Seine-Saint-Denis, France) and 10–30 mg of epirubicin at a specific ratio for feeding artery embolization. For lesions with rich blood supply, gelatin sponge granules or embolic microspheres were added in the mid-phase of the procedure, and embolization was stopped when blood flow stagnated. After the procedure, CBCT and 3D reconstruction were performed to assess the lipiodol deposition in the tumor; additional embolization was done if needed until a satisfactory embolization effect was achieved, with details available in previous studies (4, 13–15).

### Percutaneous RFA procedure

The pRFA procedure was performed within 4 weeks after TACE. The commonly used guidance methods included routine ultrasound combined with computed tomography (CT) or ultrasound combined with CBCT. The equipment selection was as follows: for pRFA, the RITA system (RITA 1500X RF generator; AngioDynamics, Manchester, GA, USA) or the LeVeen system (LeVeen™ SuperSlim™ Needle Electrode System, Boston Scientific) were adopted, and the ablation power and duration were set with reference to the parameter recommendations provided by the respective manufacturers.

**Figure 2:**
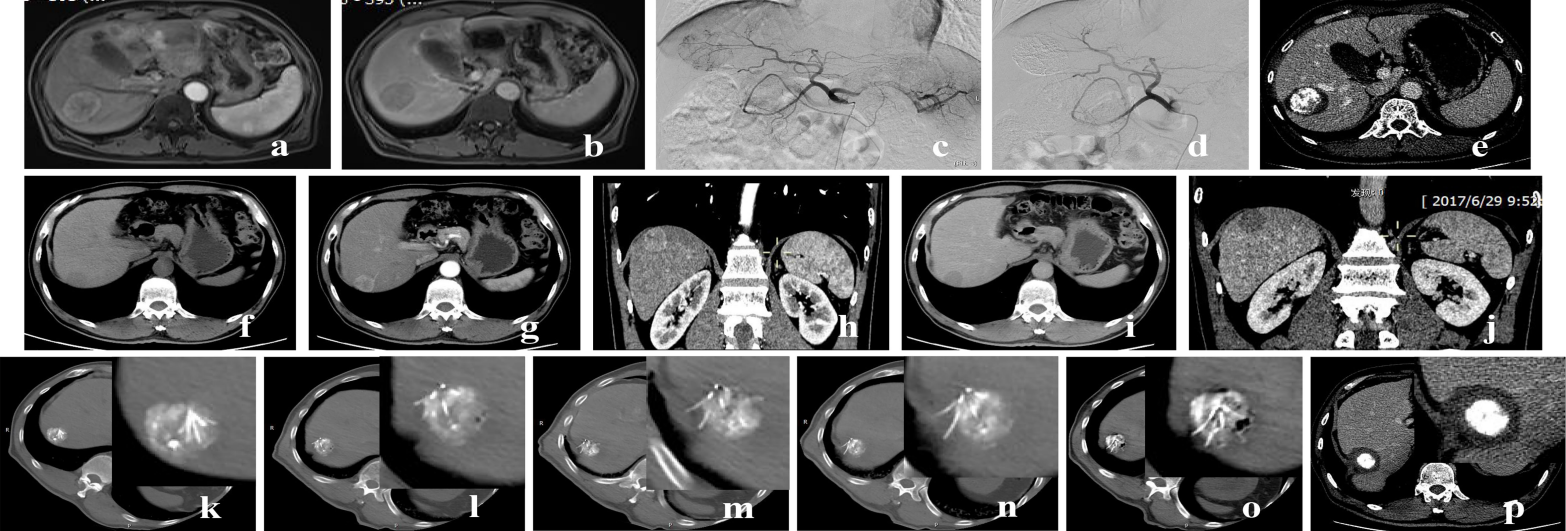
Images of a 63-year-old male with a single hepatocellular carcinoma (HCC) nodule in segment 8 (S8) of the liver, with a maximum diameter about 4.5 cm. **(a, b)** Preoperative MRI showed obvious enhancement in the late arterial phase, washout in the venous phase. **(c, d)** During transarterial chemoembolization (TACE), lesion staining was observed, and angiography after super-selective embolization showed disappearance of the staining. **(e)** Postoperative follow-up CT showed satisfactory iodized oil deposition, no tumor enhancement, and clear ablation margins. Images of another 63-year-old male patient with a single HCC nodule in segment 7 (S7) of the liver, with a maximum diameter of 3 cm. **(f–j)** Preoperative contrast-enhanced computed tomography (CT) showed obvious enhancement of the nodule in the late arterial phase and washout in the venous phase (consistent with the typical imaging features of HCC). **(k–o)** After TACE was performed for the lesion, percutaneous radiofrequency ablation (pRFA) was performed under the guidance of ultrasound combined with CT. During the procedure, the lesion was accurately targeted, and multi-point needle placement was conducted to achieve complete ablation. **(p)** Postoperative follow-up CT showed satisfactory lipiodol deposition in the tumor bed, no contrast enhancement of the tumor (indicating complete necrosis), and a clear ablation margin.

The entire treatment process was conducted under general intravenous anesthesia and electrocardiographic monitoring, following the steps below: First, local infiltration anesthesia was completed with 1% local lidocaine solution; then, the ablation needle was inserted into the tumor under real-time ultrasound guidance. After the operator confirmed the satisfactory position of the needle via CT/CBCT, the needle’s sub-needles were deployed for ablation. Post-ablation, CT/CBCT was used to evaluate the safety margin of tumor ablation; if necessary, the needle position was adjusted and ablation was repeated until the margin met the required standard for tumor ablation (**Figure 2**). Specific operational details can be found in our previous studies (4, 13–15).

### Variable collection and follow-up

The general data collected in this study were essentially the same as those in previous studies (15). The primary endpoint was PFS, defined as the time from the initiation of pRFA to the date of either tumor progression or death from any cause; the definition of PFS referred to our previous studies (4, 13). The secondary endpoint was defined as postoperative complications, which included bleeding, local hematoma, local skin infection, pain, and gastrointestinal reactions. All these symptoms were classified in accordance with the guidelines of the Society of Interventional Radiology (16). Major complications were defined as any adverse event requiring additional treatment, such as escalation of care, prolonged hospital stay, mortality resulting from the complication, or disability caused by the complication.

Within the first year after the pRFA procedure, patients underwent follow-up once every 3 months; after 1 year, the follow-up interval was adjusted to 3–6 months. During each follow-up visit, in addition to evaluating the patients’ clinical symptoms, the results of laboratory tests, contrast-enhanced CT scans or magnetic resonance imaging (MRI) examinations were recorded, and the patients’ tumor progression and survival status were also documented. Specific details can be found in our previous studies (4, 13–15).

### Statistical analysis

Data processing and analyses were performed using R version 4.3.0 and the Storm Statistical Platform (available at www.medsta.cn/software) Continuous variables with a normal distribution were expressed as mean ± standard deviation (SD), and comparisons between groups were conducted using the independent samples t-test (for two groups) or one-way analysis of variance (ANOVA; for three or more groups). Continuous variables with a non-normal distribution were analyzed using non-parametric tests (consistent with subsequent Wilcoxon rank-sum test description). Categorical variables were presented as n (%), and comparisons between groups were performed using the chi-square (χ²) test; Fisher’s exact test was used when the expected frequency of any cell in the contingency table was < 5. Baseline characteristics and outcomes were compared between the two groups as follows: categorical variables were analyzed using the χ² test or Fisher’s exact test (as appropriate), and continuous variables were analyzed using the Student’s t-test (for normally distributed data) or Wilcoxon rank-sum test (for non-normally distributed data).

PFS was estimated using the Kaplan-Meier method, and differences in PFS between groups were compared using the log-rank test. Univariable and multivariable Cox proportional-hazards regression analyses were performed to identify risk factors for PFS. Baseline variables that were clinically relevant or showed a significant association with PFS in the univariable analysis were included in the multivariable Cox regression model. To ensure parsimony of the final model, variables for inclusion were carefully selected based on the number of outcome events (to avoid overfitting). A two-sided *p* value < 0.05 was considered statistically significant.

## Results

### Baseline characteristics of the patients

A total of 118 patients received TACE-pRFA as initial treatment for very early/early-stage HCC; among them, 90 patients were in the ≤ 3 cm group and 28 patients were in the > 3 cm group. No significant differences were observed between the two groups in terms of age, sex ratio, maximum tumor diameter, cirrhosis rate, tumor etiology, Child–Pugh class, ALBI grade, ECOG PS, AFP level, or the proportions of hypertension, diabetes, smoking, and alcohol abuse (**Table 1**).

**Table 1:** Baseline characteristics of the 118 patients who received TACE-pRFA as the first-line option for hepatocellular carcinoma.

Variables	Total (n = 118)	Maximum tumor diameter		*p*-value
		≤3 cm (n = 90)	>3 cm (n = 28)	
Age, years	59.47 ± 11.82	58.39 ± 11.09	62.93 ± 13.56	0.076
Sex, n (%)				0.257
female	26 (22.03)	22 (24.44)	4 (14.29)	
male	92 (77.97)	68 (75.56)	24 (85.71)	
Hypertension, n (%)				0.438
no	86 (72.88)	64 (71.11)	22 (78.57)	
yes	32 (27.12)	26 (28.89)	6 (21.43)	
Diabetes, n (%)				0.401
no	97 (82.2)	72 (80.00)	25 (89.29)	
yes	21 (17.8)	18 (20.00)	3 (10.71)	
Smoking, n (%)				0.883
no	66 (55.93)	50 (55.56)	16 (57.14)	
yes	52 (44.07)	40 (44.44)	12 (42.86)	
Alcohol, n (%)				0.974
no	84 (71.19)	64 (71.11)	20 (71.43)	
yes	34 (28.81)	26 (28.89)	8 (28.57)	
Whether TACE is contemporaneous with pRFA, n (%)				0.217
no	96 (81.36)	71 (78.89)	25 (89.29)	
yes	22 (18.64)	19 (21.11)	3 (10.71)	
Location of tumor, n (%)				0.505
left lobe	31 (26.27)	25 (27.78)	6 (21.43)	
right lobe	87 (73.73)	65 (72.22)	22 (78.57)	
BCLC staging, n (%)				<.001
0	61 (51.69)	61 (67.78)	0 (0.00)	
A	57 (48.31)	29 (32.22)	28 (100.00)	
Child-Pugh class, n (%) †				0.281
A	102 (88.69)	76 (86.36)	26 (96.30)	
B	13 (11.30)	12 (13.64)	1 (3.70)	
ALBI class, n (%) †				0.688
1	54 (46.15)	42 (47.19)	12 (42.86)	
2	63 (53.85)	47 (52.81)	16 (57.14)	
Cirrhosis, n (%)				0.150
no	43 (36.44)	36 (40.00)	7 (25.00)	
yes	75 (63.56)	54 (60.00)	21 (75.00)	
ECOG PS, n (%)				0.266
0	109 (92.37)	85 (94.44)	24 (85.71)	
1	9 (7.63)	5 (5.56)	4 (14.29)	
Liver disease type, n (%)				0.741
no	21 (17.80)	16 (17.78)	5 (17.86)	
hepatitis B/C virus	93 (78.81)	70 (77.78)	23 (82.14)	
others	4 (3.39)	4 (4.44)	0 (0.00)	
AFP, n (%)				0.490
≤ 20 ng/ml	78 (66.10)	61 (67.78)	17 (60.71)	
>20 ng/ml	40 (33.90)	29 (32.22)	11 (39.29)	
Serum albumin, g/L	39.05 ± 4.91	39.26 ± 5.17	38.40 ± 4.00	0.421
Creatinine, μmol/L	78.13 ± 14.05	78.75 ± 14.53	76.13 ± 12.43	0.392
Hemoglobin, g/L	138.00 (129.00 - 149.00)	140.00 (129.00 - 151.00)	134.00 (127.00 - 145.25)	0.199
White cell count, ×10^9^/L	4.90 (3.70 - 5.70)	5.00 (3.70 - 5.70)	4.60 (3.22 - 5.70)	0.460
Platelet count, ×10^9^/L	120.00 (88.00 - 155.00)	115.00 (89.00 - 151.00)	124.00 (84.75 - 171.25)	0.598
ALT, IU/L	21.00 (14.00 - 30.00)	21.00 (15.00 - 28.00)	19.50 (13.00 - 36.50)	0.602
AST, IU/L	26.00 (22.00 - 36.00)	26.00 (22.00 - 36.00)	24.50 (20.75 - 36.75)	0.578
Total bilirubin, umol/L	15.70 (12.00 - 22.00)	15.70 (12.00 - 20.80)	15.90 (12.05 - 22.10)	0.975
Alkaline phosphatase, IU/L	74.00 (63.00 - 89.00)	72.00 (62.00 - 92.00)	78.00 (67.50 - 87.25)	0.466
International standardized ratio	1.09 (1.01 - 1.19)	1.08 (1.00 - 1.19)	1.11 (1.03 - 1.21)	0.413
Prothrombin time, s	11.80 (11.25 - 13.05)	11.80 (11.20 - 13.22)	12.30 (11.30 - 12.85)	0.966

Data are presented as n (%), median (Q1, Q3), or mean (± SD).

TACE, transarterial chemoembolization; pRFA, percutaneous radiofrequency ablation; BCLC, Barcelona Clinic Liver Cancer; ALBI, albumin bilirubin; ECOG PS, Eastern Cooperative Oncology Group performance status; AFP, alpha-fetoprotein; ALT, alanine transaminase; AST, aspartate aminotransferase.

† Missing data.

### Tumor progression, prognostic factors of PFS, and salvage treatments

The median follow-up duration was 31.0 months (95% confidence interval [CI], 24.8–37.2) for the total cohort, and the median PFS was 35.0 months (95% CI, 24.3–45.7) for the entire group.

In the total cohort, 4 of 118 patients (3.4%) developed local tumor progression (LTP): 2 of 90 patients (2.2%) in the ≤ 3 cm group and 2 of 28 patients (7.1%) in the > 3 cm group (*p* = 0.238). Intrahepatic distant recurrence (IDR) occurred in 54 of 118 patients (45.8%): 39 of 90 patients (43.3%) in the ≤ 3 cm group and 15 of 28 patients (53.6%) in the > 3 cm group (*p* = 0.342). Portal vein tumor thrombus (PVTT) was observed in 6 of 118 patients (5.1%): 2 of 90 patients (2.2%) in the ≤ 3 cm group and 4 of 28 patients (14.3%) in the > 3 cm group (*p* = 0.041). Similarly, extrahepatic recurrence occurred in 4 of 118 patients (3.4%): 3 of 90 patients (3.3%) in the ≤ 3 cm group and 1 of 28 patients (3.6%) in the > 3 cm group (*p* = 1.000) (**Table 2**).

**Table 2:** Tumor recurrence patterns after TACE-pRFA.

Variable	Total (n = 118)	≤ 3 cm (n = 90)	> 3 cm (n = 28)	*p*-value
Tumor events after TACE-pRFA				
LTP, n (%)	4 (3.39)	2 (2.22)	2 (7.14)	0.238
IDR, n (%)	54 (45.76)	39 (43.33)	15 (53.57)	0.342
PVTT, n (%)	6 (5.08)	2 (2.22)	4 (14.29)	0.041
Extrahepatic recurrence, n (%)	4 (3.39)	3 (3.33)	1 (3.57)	1.000

TACE-pRFA, transarterial chemoembolization plus percutaneous radiofrequency ablation; LTP, local tumor progression; IDR, intrahepatic distant recurrence; PVTT, portal vein tumor thrombus.

**Figure 3:**
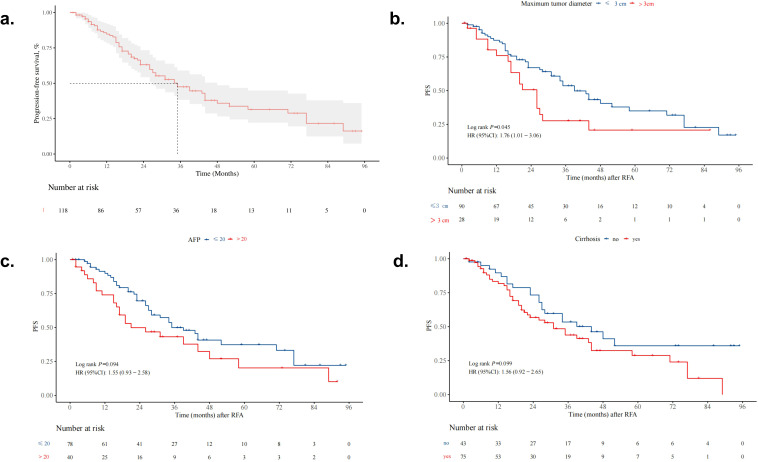
Kaplan–Meier curves for progression-free survival (PFS). **(a)** Kaplan–Meier curve of PFS in the entire study cohort. **(b)** Kaplan–Meier curve comparing PFS between the ≤ 3 cm group and the > 3 cm group (*p* = 0.045). **(c)** Kaplan–Meier curve comparing PFS between two groups stratified by alpha-fetoprotein (AFP) level (> 20 ng/mL vs. ≤ 20 ng/mL; *p* = 0.094). **(d)** Kaplan–Meier curve comparing PFS between two groups stratified by the presence of cirrhosis (cirrhosis-present vs. cirrhosis-absent; *p* = 0.099).

The 1-, 3-, and 5-year cumulative PFS rates in the whole cohort were 84.7% (95% CI, 78.1–91.9%), 47.5% (95% CI, 38.2–59.0%), and 31.5% (95% CI, 22.2–44.9%), respectively (**Figure 3a**). The cumulative PFS rate was significantly higher in the ≤ 3 cm group than in the > 3 cm group (median PFS: 39.0 months [95% CI, 34.0–71.0] vs. 26.0 months [95% CI, 17.0–44.0]; p = 0.045) (**Figure 3b**). However, no significant difference in cumulative PFS rate was observed between the two AFP groups (median PFS: 21.0 months [95% CI, 17.0–58.0] in the > 20 ng/mL group vs. 35.0 months [95% CI, 28.0–77.0] in the ≤ 20 ng/mL group; p = 0.094) (**Figure 3c**) or between the two cirrhosis groups (median PFS: 44.0 months [95% CI, 27.0–not reached] in the cirrhosis-absent group vs. 31.0 months [95% CI, 21.0–44.0] in the cirrhosis-present group; p = 0.099) (**Figure 3d**).

Univariable and multivariable Cox proportional-hazards regression analyses showed that maximum tumor diameter (hazard ratio [HR], 2.87; 95% CI, 1.15–7.12; *p* = 0.023) was an independent prognostic factor for PFS after TACE-pRFA (**Table 3**). Salvage treatments for initial tumor progression are presented in **Table 4**.

**Table 3:** Univariate and multivariate analysis of factors associated with PFS.

Variables	Univariate analysis		Multivariate analysis	
	HR (95%CI)	*p*-value	HR (95%CI)	*p*-value
Age	1.01 (0.99 - 1.04)	0.213		
Sex				
female	Ref			
male	0.51 (0.28 - 0.92)	0.026	0.99 (0.96 - 1.03)	0.749
Hypertension				
no	Ref			
yes	0.99 (0.58 - 1.69)	0.962		
Diabetes				
no	Ref			
yes	1.35 (0.73 - 2.49)	0.341		
Smoking				
no	Ref			
yes	0.91 (0.55 - 1.50)	0.700		
Alcohol				
no	Ref			
yes	1.17 (0.69 - 1.97)	0.569		
Whether TACE is contemporaneous with pRFA				
no	Ref			
yes	0.99 (0.51 - 1.91)	0.966		
Location of tumor				
left	Ref			
right	0.64 (0.37 - 1.11)	0.112		
BCLC staging				
0	Ref			
A	1.40 (0.85 - 2.30)	0.188		
Child Pugh class				
A	Ref			
B	1.77 (0.83 - 3.74)	0.138		
ALBI class				
1	Ref			
2	1.35 (0.81 - 2.23)	0.246		
Cirrhosis				
no	Ref		Ref	
yes	1.56 (0.92 - 2.65)	0.099	0.52 (0.12 - 2.25)	0.381
ECOG PS				
0	Ref			
1	1.30 (0.47 - 3.62)	0.618		
Liver disease type				
no	Ref			
hepatitis B/C virus	1.19 (0.56 - 2.51)	0.647		
others	0.46 (0.06 - 3.71)	0.468		
AFP				
≤ 20 ng/ml	Ref		Ref	
>20 ng/ml	1.55 (0.93 - 2.58)	0.094	1.15 (0.43 - 3.05)	0.779
Maximum tumor diameter				
≤3 cm	Ref		Ref	
>3 cm	1.76 (1.01 - 3.06)	0.045	2.87 (1.15 - 7.12)	0.023
Prothrombin time	1.10 (0.93 - 1.29)	0.257		
International standardized ratio	1.30 (0.23 - 7.17)	0.766		
Creatinine	0.99 (0.97 - 1.01)	0.22		
Alkaline phosphatase	1.00 (0.99 - 1.01)	0.922		
Total bilirubin	0.99 (0.96 - 1.02)	0.346		
Serum albumin	0.95 (0.90 - 0.99)	0.025	1.97 (0.77 - 5.04)	0.156
AST	0.99 (0.98 - 1.01)	0.310		
ALT	0.99 (0.98 - 1.01)	0.206		
Platelet count	1.00 (0.99 - 1.00)	0.528		
White cell count	0.93 (0.81 - 1.07)	0.316		
Hemoglobin	0.99 (0.97 - 0.99)	0.030	0.96 (0.87 - 1.05)	0.379

PFS, progression-free survival; HR, hazard ratio; CI, confidence interval; TACE, transarterial chemoembolization; pRFA, percutaneous radiofrequency ablation; BCLC, Barcelona Clinic Liver Cancer; ALBI, albumin bilirubin; ECOG PS, Eastern Cooperative Oncology Group performance status; AFP, alpha-fetoprotein; ALT, alanine transaminase; AST, aspartate aminotransferase.

**Table 4:** Second-line treatments.

Type of treatments	Number of patients
TACE-pRFA	n=23
TACE/TAI	n=15
pRFA	n=2
TACE-pRFA+ systemic therapy	n=1
Systemic therapy	n=1
Support therapy	n=4
Unknown	n=4
No	n=68

TACE-pRFA, transarterial chemoembolization plus percutaneous radiofrequency ablation; TAI, transarterial infusion.

### Complications

No procedure-related mortality, disability, or major complications occurred in either group. In the total cohort, 80 of 118 patients (67.8%) reported discomfort: 63 of 90 patients (70.0%) in the ≤ 3 cm group and 17 of 28 patients (60.7%) in the > 3 cm group. There was no statistically significant difference in the incidence of discomfort between the two groups (*p* = 0.358) (**Table 5**). All complications and reported discomfort were successfully managed with appropriate treatment and resolved gradually.

**Table 5:** The patient’s complications requiring medication after treatment.

Variable	Total (n = 118)	≤3 cm (n = 90)	>3 cm (n = 28)	*p-*value
Complications, n (%)	80 (67.8)	63 (70.00)	17 (60.71)	0.358
elevated ALT, n (%)	58 (49.15)	45 (50.00)	13 (46.43)	
elevated AST, n (%)	71 (60.17)	57 (63.33)	14 (50.00)	
elevated total bilirubin, n (%)	39 (33.05)	34 (37.78)	5 (17.86)	
fever, n (%)	1 (0.85)	1 (1.11)	0 (0.00)	
pain, n (%)	23 (19.49)	16 (17.78)	7 (25.00)	
vomiting, n (%)	2 (3.45)	2 (4.76)	0 (0.00)	

ALT, alanine transaminase; AST, aspartate aminotransferase.

## Discussion

In this study, we found that the 1-, 3-, and 5-year cumulative PFS rates in the whole cohort were 84.7%, 47.5%, and 31.5%, respectively. Univariable and multivariable analyses revealed that maximum tumor diameter was the only independent prognostic factor for PFS after TACE-pRFA. Treatment-related discomfort was comparable between the ≤ 3 cm group and the > 3 cm group. Moreover, we observed that certain patterns of HCC recurrence or progression—such as PVTT—occurred more frequently in patients with tumors > 3 cm than in those with tumors ≤ 3 cm, although no significant differences were found in intrahepatic or extrahepatic progression. As tumor size increases, the rate of adjacent microvascular invasion and micrometastasis rises, which is thought to contribute significantly to PVTT development after ablation (17). Nearly one-third of tumors ≤ 3 cm have undetectable micrometastases or satellite nodules; while most satellite lesions are located within 1 cm of the primary tumor, approximately 17% are more than 1 cm away (18). This study therefore raises an important question regarding the optimal ablation margin: Is a ≥ 3 mm safe ablation margin truly “safe”? (19).

However, achieving a safe ablative margin is practically challenging for HCC > 3 cm. Thus, the combination of pRFA with TACE is increasingly being investigated for HCC treatment (3–9)—a factor that likely contributed to the relatively satisfactory efficacy and safety observed in this study. This outcome is also attributable to the continuous improvement and maturation of current TACE and pRFA techniques. pRFA is recommended by the BCLC guidelines as a first-line treatment for very early/early-stage HCC due to its minimal invasiveness, cost-effectiveness, and favorable safety profile, and it can achieve satisfactory curative effects for HCC ≤ 3 cm (1, 2). However, pRFA has obvious limitations, especially for tumors > 3 cm: the “heat sink effect” caused by abundant tumor blood supply easily leads to incomplete ablation, and the difficulty in obtaining a sufficient safe ablation margin increases the risk of LTP and IDR (3, 5). In contrast, TACE-pRFA combines the advantages of TACE and pRFA: (1) additional survival benefit from chemoembolization; (2) TACE performed before ablation aids in lesion localization (10)—specifically, lipiodol or microsphere deposition allows surgeons to clearly identify lesion locations under multi-modality imaging guidance (ultrasound/CT/CBCT/DSA), thereby facilitating positioning; (3) lipiodol or microsphere deposition within the tumor enhances heat conduction, promoting complete tumor necrosis; (4) TACE reduces the inherent “heat sink effect” of thermal ablation (10, 20) by blocking abundant blood flow in and around the tumor; (5) for micrometastases or satellite nodules not detected by preoperative imaging (e.g., contrast-enhanced CT, MRI, ultrasound), arteriography during TACE enables early identification, and timely intervention for these lesions may improve patient prognosis; (6) strict adherence to a safe ablation margin (i.e., ≥ 10 mm) (18). Despite the satisfactory efficacy and safety observed in this study, tumor progression or recurrence—especially IDR—cannot be ignored, and timely intervention is required to improve prognosis. In this study, although all patients received TACE-pRFA for their index lesions, it remains unclear whether recurrent lesions detected during postoperative follow-up are due to multicentric origin or intrahepatic metastasis (the latter typically occurs earlier). Prognostically, even if progression or recurrence occurs after first-line treatment, favorable outcomes can still be achieved with salvage surgery or locoregional therapy (21). In contrast, intrahepatic metastases arise from clonally related tumor cells, and such tumors exhibit more aggressive biological behavior; even with intensive treatment, their prognosis remains poor (22).

Several studies have validated the use of TACE-pRFA for early or very early-stage HCC. A previous single-institution study (7) reported a median PFS of 34.9 months (95% CI, 29.4–40.4), which is comparable to the median PFS of 35.0 months (95% CI, 24.3–45.7) observed in the present study, for tumors > 3 cm, a median PFS of 26.0 months was obtained, which is superior to the reported efficacy of pRFA alone for tumors > 3 cm (median PFS usually < 20 months) (4, 23). Another recent single-institution study (23) included 538 patients with single medium-sized (3–5 cm) HCC treated between 2000 and 2016; these patients received first-line TACE-pRFA (n=109), TACE alone (n=314), or pRFA alone (n=115). During follow-up in that study (23), disease recurrence was detected in 75 of the 109 patients (68.8%) who received TACE-pRFA. In contrast, we observed recurrence in only 68 of 118 patients (57.6%) in our cohort. Notably, in study (23), the first recurrence events included LTP in 31 cases (28.4%), IDR in 41 cases (37.6%), and simultaneous LTP and IDR in 3 cases (2.8%)—a pattern inconsistent with our findings and those of several recent studies (4, 24). This discrepancy highlights the need for future research focused on the diagnosis and prediction of IDR (a common HCC recurrence pattern) and more effective monitoring of this patient population. Whether using state-of-the-art multi-omics analysis or other high-throughput big data approaches, the goal should be to maximize the prognosis of patients with HCC.

Given its minimal invasiveness and low incidence of postoperative complications, pRFA is recommended by major international guidelines (1). From a safety perspective, TACE-pRFA exhibited a favorable safety profile, with major adverse events responsive to treatment and no associated mortality or disability. Cao et al. (4) reported major complications in 15 of 234 patients (6.4%)—including 2 cases of hepatic infarction, 4 cases of portal vein thrombosis, 1 case of biloma, 1 case of liver abscess, 2 cases of bleeding, and 5 additional cases of portal vein thrombosis (note: original text duplicated “portal vein thrombosis”; corrected to clarify total 15 cases). In contrast, Chu et al. (20) reported a lower major complication rate (approximately 0.9%), with 1 patient experiencing intraperitoneal bleeding. In the present study, no procedure-related mortality, disability, or major complications occurred in either group. Instead, 80 of 118 patients (67.8%) reported discomfort: 63 of 90 patients (70.0%) in the ≤ 3 cm group and 17 of 28 patients (60.7%) in the > 3 cm group. No statistically significant difference in discomfort incidence was observed between the two groups. All complications or discomfort resolved or significantly improved with symptomatic treatment. The disadvantage of TACE-pRFA compared with pRFA alone is that it requires two sequential procedures, which may slightly increase the short-term discomfort of patients, but our study showed that the incidence of treatment-related discomfort was comparable between the two tumor size groups, and no major complications or mortality occurred, indicating that the safety of TACE-pRFA is still controllable.

TACE is a classic locoregional therapy for intermediate-stage HCC, but its efficacy for early-stage HCC is limited by incomplete tumor necrosis. Studies have shown that TACE alone for early-stage single HCC ≤ 5 cm has a higher recurrence rate (usually > 70%) and shorter PFS (median PFS: 25–30 months) (7, 23), which is inferior to TACE-pRFA in our study (recurrence rate 57.6%, median PFS 35.0 months). The core advantage of TACE-pRFA over TACE alone is that pRFA can directly ablate the tumor lesion after TACE embolization, achieving more thorough tumor necrosis and reducing the risk of residual tumor cells leading to recurrence (6, 9). In addition, TACE alone is more likely to cause damage to normal liver tissue due to non-selective embolization, while TACE-pRFA can precisely locate the tumor through multi-modal imaging guidance after TACE, reducing the injury to surrounding normal liver tissue. The disadvantage of TACE-pRFA compared with TACE alone is the relatively higher technical requirements and longer treatment cycle, which may increase the economic burden of patients to a certain extent.

Surgical resection is the gold standard for curative treatment of early-stage HCC, which can achieve complete resection of the tumor and surrounding potentially invasive tissues, and has a clear advantage in long-term overall survival (OS) for patients with good liver function (1, 21). However, surgical resection has obvious limitations: it is highly invasive, has a longer postoperative recovery period, and is not suitable for patients with poor liver function (e.g., Child-Pugh class B/C), multiple comorbidities, or poor performance status. In contrast, TACE-pRFA has the advantages of minimal invasiveness, fast recovery, and good tolerability, making it an ideal alternative treatment for early-stage HCC patients who are not suitable for surgical resection (e.g., elderly patients, patients with compromised liver function, or those who refuse surgery) (7, 11). Our study included patients with Child-Pugh class A or B and ECOG PS 0-1, and all patients completed TACE-pRFA treatment successfully, confirming its good applicability. The main disadvantage of TACE-pRFA compared with surgical resection is that it is difficult to achieve complete ablation of large tumors or tumors with complex locations, and the long-term OS may be slightly inferior to surgical resection for eligible patients (23).

Stereotactic body radiation therapy (SBRT) is a non-invasive treatment modality that can deliver high-dose radiation to the tumor with high precision, and it is suitable for small HCC that is not suitable for pRFA or surgery, especially for tumors in special locations (e.g., perivascular, subcapsular) (20). However, SBRT has the risk of radiation-induced liver injury, and its long-term efficacy and safety for early-stage HCC still need more large-sample studies to verify. Microwave ablation (MWA) is another thermal ablation technique similar to pRFA, which has the advantages of faster ablation speed and larger ablation range, but it is more likely to cause adjacent tissue injury, and the efficacy for tumors > 3 cm is still controversial (3, 6). Compared with SBRT and MWA, TACE-pRFA has a more mature clinical application basis, and its efficacy and safety have been fully verified by numerous studies (4, 7, 15); moreover, TACE-pRFA can flexibly adjust the treatment strategy according to the tumor blood supply and size, which has better individual adaptability.

Based on the above comparisons, TACE-pRFA should be positioned as an important first-line locoregional combination therapy for very early/early-stage single HCC ≤ 5 cm, especially for the following patient populations: (1) Patients with HCC > 3 cm and ≤ 5 cm, for whom pRFA alone is prone to incomplete ablation and TACE alone has poor curative effect; (2) Patients with very early/early-stage HCC who are not suitable for surgical resection due to poor liver function, multiple comorbidities, or old age; (3) Patients with HCC in special locations (e.g., subcapsular, periportal) where a sufficient safe ablation margin is difficult to obtain by pRFA alone, and TACE can assist in improving the ablation effect. For patients with HCC ≤ 3 cm and good liver function, pRFA alone can still be used as the first choice; for patients with eligible liver function and tumor conditions, surgical resection remains the preferred curative treatment; SBRT and MWA can be used as alternative treatment options for patients who are not suitable for TACE-pRFA.

This study has several limitations. First, this study is a single-center, small-sample analysis, and its main limitation lies in its retrospective nature, which inevitably introduces selection bias. Second, the definition of early-stage HCC in this study was restricted to single tumors > 2 cm, excluding patients with 2–3 lesions each ≤ 3 cm (a subset also classified as early-stage HCC in some guidelines). Third, detailed data on the time interval between TACE and pRFA were not analyzed—only a simple distinction between contemporaneous and non-contemporaneous treatment was made. The optimal time interval between TACE and pRFA remains controversial, and further studies are needed to address this question (23).

## Conclusion

In conclusion, TACE combined with pRFA is safe for very early/early-stage single HCC ≤ 5 cm. Maximum tumor diameter is an independent prognostic factor for PFS, while treatment-related complications are comparable between tumors ≤ 3 cm and > 3 cm. Thus, further studies are warranted to explore the optimal treatment modality for single HCC > 3 cm.

## Data Availability

The original contributions presented in the study are included in the article/supplementary material. Further inquiries can be directed to the corresponding author.

## Glossary

AFP: alpha-fetoprotein
ALBI: albumin bilirubin
ALT: alanine transaminase
AST: aspartate aminotransferase
BCLC: Barcelona Clinic Liver Cancer
CI: confidence interval
ECOG PS: Eastern Cooperative Oncology Group performance status
HR: hazard ratio
IDR: intrahepatic distant recurrence
LTP: local tumor progression
PFS: progression-free survival
pRFA: percutaneous radiofrequency ablation
PVTT: portal vein tumor thrombus
TACE: transarterial chemoembolization
TAI: transarterial infusion.

## References

[1] Forner A Reig M Bruix J . Hepatocellular carcinoma. Lancet. (2018) 391:1301–14. doi: 10.1016/S0140-6736(18)30010-2, PMID: 29307467

[2] Lee DH Lee JM Lee JY Kim SH Yoon JH Kim YJ . Radiofrequency ablation of hepatocellular carcinoma as first-line treatment: long-term results and prognostic factors in 162 patients with cirrhosis. Radiology. (2014) 270:900–9. doi: 10.1148/radiol.13130940, PMID: 24475823

[3] Han K Kim JH Kim GH Kim JH Kim SY Park SH . Radiofrequency ablation of subcapsular versus nonsubcapsular hepatocellular carcinomas ≤ 3 cm: analysis of long-term outcomes from two large-volume liver centers. Eur Radiol. (2023) 34(3):1578–86. doi: 10.1007/s00330-023-10165-6, PMID: 37646813

[4] Cao S Lyu T Fan Z Guan H Song L Tong X . Long-term outcome of percutaneous radiofrequency ablation for periportal hepatocellular carcinoma: tumor recurrence or progression, survival and clinical significance. Cancer Imaging. (2022) 22:2. doi: 10.1186/s40644-021-00442-2, PMID: 34983650 PMC8725335

[5] Sugimori K Morimoto M Shirato K Kokawa A Tomita N Saito T . Radiofrequency ablation in a pig liver model: effect of transcatheter arterial embolization on coagulation diameter and histologic characteristics. Hepatol Res. (2002) 24:164. doi: 10.1016/s1386-6346(02)00030-x, PMID: 12270746

[6] Kim HI An J Han S Shim JH . Loco-regional therapies competing with radiofrequency ablation in potential indications for hepatocellular carcinoma: a network meta-analysis. Clin Mol Hepatol. (2023) 29:1013–28. doi: 10.3350/cmh.2023.0131, PMID: 37403319 PMC10577337

[7] Hyun D Cho SK Shin SW Park KB Park HS Choo SW . Early stage hepatocellular carcinomas not feasible for ultrasound-guided radiofrequency ablation: comparison of transarterial chemoembolization alone and combined therapy with transarterial chemoembolization and radiofrequency ablation. Cardiovasc Intervent Radiol. (2016) 39:417–25. doi: 10.1007/s00270-015-1194-0, PMID: 26246215

[8] Hyun D Cho SK Shin SW Rhim H Koh KC Paik SW . Treatment of small hepatocellular carcinoma (≤2 cm) in the caudate lobe with sequential transcatheter arterial chemoembolization and radiofrequency ablation. Cardiovasc Intervent Radiol. (2016) 39:1015–22. doi: 10.1007/s00270-016-1314-5, PMID: 26975761

[9] Lee MW Kim YJ Park SW Yu NC Choe WH Kwon SY . Biplane fluoroscopy-guided radiofrequency ablation combined with chemoembolisation for hepatocellular carcinoma: initial experience. Br J Radiol. (2011) 84:691–7. doi: 10.1259/bjr/27559204, PMID: 21750136 PMC3473436

[10] Yang BS Liu LX Yuan M Hou YB Li QT Zhou S . Multiple imaging modality-guided radiofrequency ablation combined with transarterial chemoembolization for hepatocellular carcinoma in special locations. Diagn Interv Radiol. (2020) 26:131–9. doi: 10.5152/dir.2019.18540, PMID: 32071022 PMC7051260

[11] Ren Y Cao Y Ma H Kan X Zhou C Liu J . Improved clinical outcome using transarterial chemoembolization combined with radiofrequency ablation for patients in Barcelona clinic liver cancer stage A or B hepatocellular carcinoma regardless of tumor size: results of a single-center retrospective case control study. BMC Cancer. (2019) 19:983. doi: 10.1186/s12885-019-6237-5, PMID: 31640620 PMC6805486

[12] Brown ZJ Tsilimigras DI Ruff SM Mohseni A Kamel IR Cloyd JM . Management of hepatocellular carcinoma: A review. JAMA Surg. (2023) 158:410–20. doi: 10.1001/jamasurg.2022.7989, PMID: 36790767

[13] Guan H Xie Y Lyu T Song L Tong X Wang J . Radiofrequency ablation with or without conventional transarterial chemoembolization for subcapsular versus nonsubcapsular hepatocellular carcinoma within Milan criteria: a propensity score-matched study. Int J Hyperthermia. (2025) 42:2452930. doi: 10.1080/02656736.2025.2452930, PMID: 40010696

[14] Xie Y Lyu T Guan H Cao S Song L Tong X . Radiofrequency ablation with or without transarterial chemoembolization for hepatocellular carcinoma meeting Milan criteria: a focus on tumor progression and recurrence patterns. Front Oncol. (2024) 14:1392495. doi: 10.3389/fonc.2024.1392495, PMID: 38751809 PMC11094263

[15] Xie Y Lyu T Song L Tong X Wang J Zou Y . TACE-assisted multi-image guided radiofrequency ablation for the treatment of single hepatocellular carcinoma ≤ 5 cm: a retrospective study. Front Oncol. (2024) 14:1347675. doi: 10.3389/fonc.2024.1347675, PMID: 38646432 PMC11026585

[16] Kim W Cho SK Shin SW Hyun D Lee MW Rhim H . Combination therapy of transarterial chemoembolization (TACE) and radiofrequency ablation (RFA) for small hepatocellular carcinoma: comparison with TACE or RFA monotherapy. Abdom Radiol (NY). (2019) 44:2283–92. doi: 10.1007/s00261-019-01952-1, PMID: 30806742

[17] Ikeda K Seki T Umehara H Inokuchi R Tamai T Sakaida N . Clinicopathologic study of small hepatocellular carcinoma with microscopic satellite nodules to determine the extent of tumor ablation by local therapy. Int J Oncol. (2007) 31:485–91. doi: 10.3892/ijo.31.3.485, PMID: 17671673

[18] Shi M Zhang CQ Zhang YQ Liang XM Li JQ . Micrometastases of solitary hepatocellular carcinoma and appropriate resection margin. World J Surg. (2004) 28:376–81. doi: 10.1007/s00268-003-7308-x, PMID: 15022021

[19] Kim YS Lee WJ Rhim H Lim HK Choi D Lee JY . The minimal ablative margin of radiofrequency ablation of hepatocellular carcinoma (> 2 and < 5 cm) needed to prevent local tumor progression: 3D quantitative assessment using CT image fusion. AJR Am J Roentgenol. (2010) 195:758–65. doi: 10.2214/AJR.09.2954, PMID: 20729457

[20] Yu Q Thapa N Karani K Navuluri R Ahmed O Van Ha T . Transarterial Radioembolization versus Transarterial Chemoembolization Plus Percutaneous Ablation for Unresectable, Solitary Hepatocellular Carcinoma of ≥3 cm: A Propensity Score-Matched Study. J Vasc Interv Radiol. (2022) 33:1570–1577.e1. doi: 10.1016/j.jvir.2022.09.005, PMID: 36100064

[21] Duan M Hao J Cui S Worthley DL Zhang S Wang Z . Diverse modes of clonal evolution in HBV-related hepatocellular carcinoma revealed by single-cell genome sequencing. Cell Res. (2018) 28:359–73. doi: 10.1038/cr.2018.11, PMID: 29327728 PMC5835770

[22] Baffy G . Decoding multifocal hepatocellular carcinoma: an opportune pursuit. Hepatobiliary Surg Nutr. (2015) 4:206–10. doi: 10.3978/j.issn.2304-3881.2014.12.05, PMID: 26151061 PMC4465603

[23] Chu HH Kim JH Yoon HK Ko HK Gwon DI Kim PN . Chemoembolization combined with radiofrequency ablation for medium-sized hepatocellular carcinoma: A propensity-score analysis. J Vasc Interv Radiol. (2019) 30:1533–43. doi: 10.1016/j.jvir.2019.06.006, PMID: 31471190

[24] Mu S Chen Q Li S Wang D Zhao Y Li X . Incomplete radiofrequency ablation following transarterial chemoembolization accelerates the progression of large hepatocellular carcinoma. J Cancer Res Ther. (2023) 19:924–32. doi: 10.4103/jcrt.jcrt_2296_22, PMID: 37675718

